# PD-L1-negative triple-negative breast cancer exhibited a remarkable response to cadonilimab: a case report

**DOI:** 10.3389/fonc.2026.1764908

**Published:** 2026-03-27

**Authors:** Baohua Chen, Yuanyuan Jia, Lingjun Meng, Nanjun Hu

**Affiliations:** 2nd Inpatient Area of Oncology, China-Japan Union Hospital of Jilin University, Changchun, Jilin, China

**Keywords:** cadonilimab, CTLA-4, immunotherapy, PD-1, triple-negative breast cancer (TNBC)

## Abstract

Breast cancer, a prevalent malignancy among women, is primarily categorized into four subtypes based on immunohistochemistry: luminal A breast cancer, luminal B breast cancer, HER2-enriched breast cancer, and triple-negative breast cancer (TNBC). Molecular targets for TNBC therapy are lacking, and TNBC is characterized by a high risk of recurrence and a poor prognosis. The advent of immune checkpoint inhibitors (ICIs), including PD-1, PD-L1, and CTLA-4 inhibitors, has led to therapeutic advances in the management of TNBC. The combination of chemotherapy and immunotherapy has been shown to improve patient outcomes and survival rates. This case primarily discusses immunotherapy combination treatments for PD-L1-negative TNBC patients, for which research remains relatively scarce. This case report focuses on a 48-year-old female patient with PD-L1-negative stage IV TNBC who exhibited a significant therapeutic response to cadonilimab, a tetravalent PD-1/CTLA-4 bispecific antibody, in combination with chemotherapy, achieving a partial response (PR), as assessed by clinical evaluation. The tumor volume was reduced by more than 75%. The patient experienced grade 3 neutropenia, grade 2 thrombocytopenia, grade 2 nausea, and grade 2 immune-related adverse events, including pruritus and hyperthyroidism. Furthermore, this report reviews the current principal treatment modalities for TNBC. Particular emphasis was placed on discussing immunotherapy combination treatments for TNBC. Additional studies with larger cohorts are warranted for comprehensive translational research on PD-L1-negative TNBC, aiming to validate the efficacy and elucidate the mechanisms of action of various ICIs. This case highlights that dual-targeted therapy may provide a promising therapeutic option for patients with PD-L1-negative TNBC.

## Introduction

Breast cancer is a heterogeneous disease comprising distinct subtypes, each characterized by unique epidemiological features. Globally, breast cancer represents approximately one-third of all malignant tumors in women and is associated with a mortality rate of approximately 15% among diagnosed cases (1). In clinical practice, breast cancer is classified into four subtypes based on immunohistochemical profiling: luminal A breast cancer, luminal B breast cancer, HER2-enriched breast cancer, and triple-negative breast cancer (TNBC). TNBC is defined by the absence of estrogen receptors (ERs), progesterone receptors (PRs), and human epidermal growth factor receptor-2 (HER2) and accounts for approximately 15–20% of primary breast tumors (2). Unlike other subtypes, specific molecular targets are lacking for TNBC therapy, and TNBC is associated with a high risk of recurrence and a poor prognosis, necessitating greater reliance on comprehensive chemotherapy (1, 3, 4).

The mainstay of traditional TNBC treatment is systemic chemotherapy, particularly regimens based on anthracyclines and taxanes. In recent years, immunotherapy has been increasingly adopted and has gradually become the standard treatment regimen for certain patient populations, such as those with PD-L1-positive tumors. Immune checkpoint inhibitors have significantly transformed the therapeutic management of various malignancies, including advanced triple-negative breast cancer (TNBC). In the KEYNOTE-355 trial, the combination of the immune checkpoint inhibitor pembrolizumab with chemotherapy demonstrated notable efficacy in patients with a PD-L1 combined positive score (CPS) ≥ 10 but not in those with a PD-L1 CPS ≥ 1 or in the intention-to-treat (ITT) population (5). The TORCHLIGHT study conducted in China reported that treatment with toripalimab combined with nab-paclitaxel significantly improved both progression-free survival (PFS) and overall survival (OS) (6). However, the efficacy of immunotherapy in patients with a CPS < 1 remains uncertain. Cadonilimab is a quadrivalent bispecific antibody (targeting PD-1 and CTLA-4 as therapeutic targets) that demonstrates significant efficacy in patients with low PD-L1 expression, such as those with gastric cancer. Here, we report a case in which a patient with distant metastatic TNBC with a CPS<1 exhibited a remarkable response to cadonilimab in combination with chemotherapy and experienced only mild immune-related adverse events (irAEs) (7–10). This case highlights that dual-targeted immunotherapy may provide a promising therapeutic option for patients with PD-L1-negative TNBC. In addition, we will examine contemporary therapeutic strategies for the management of TNBC.

## Case presentation

**Figure 1:**
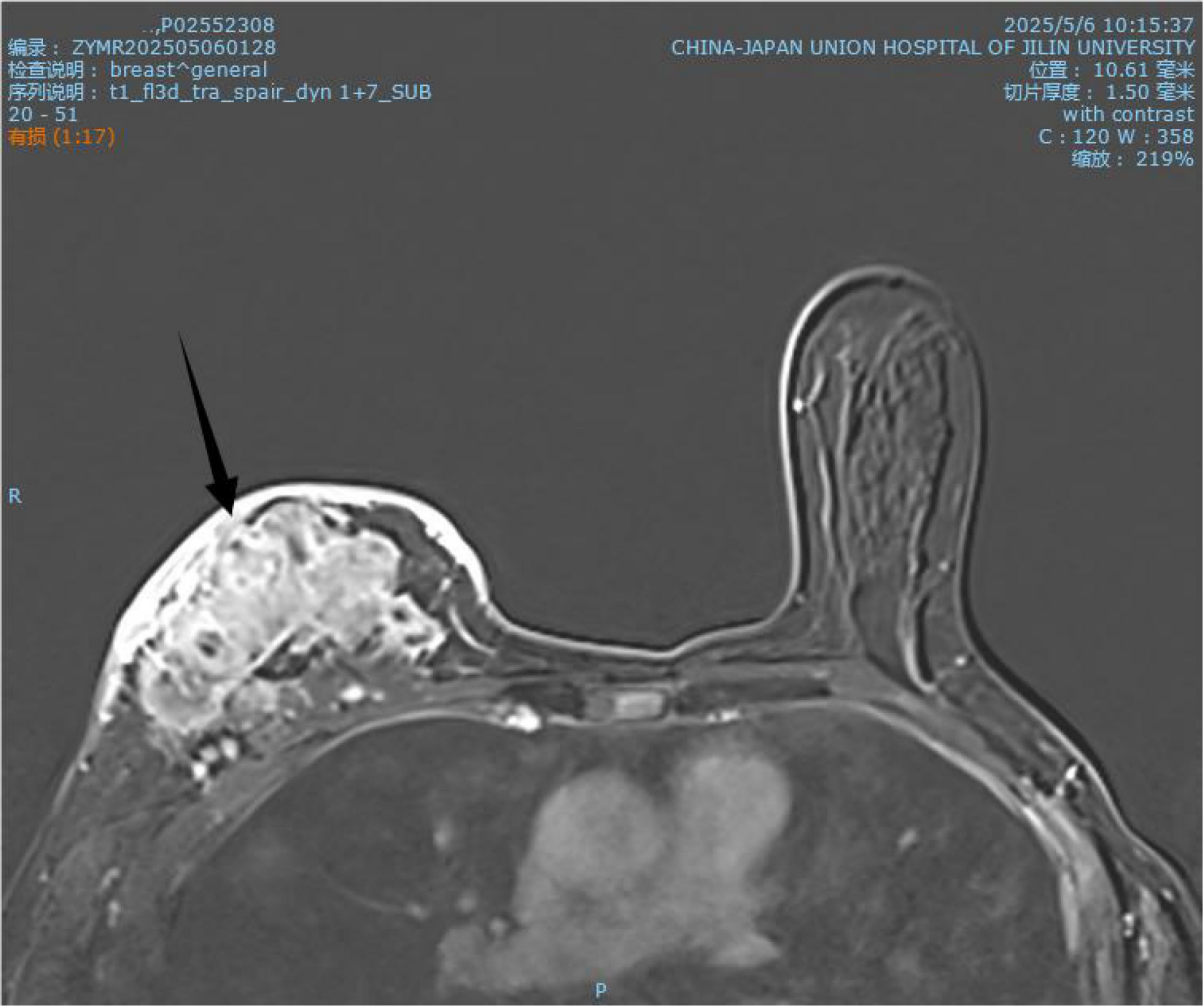
MR image from May 2025.

A 48-year-old woman presented in November 2024 with a palpable mass in her right breast. Physical examination revealed a discrete mass measuring approximately 3.0 cm, accompanied by localized peau d’orange changes on the right breast and palpable, mobile ipsilateral axillary lymph nodes measuring 2.0 cm. Mammography and ultrasound imaging confirmed the presence of a 3.0-cm mass. Biopsies of the breast mass and axillary lymph nodes revealed invasive triple-negative ductal carcinoma, which differs from traditional breast cancer, characterized by estrogen receptor (ER) negativity (0%), progesterone receptor (PR) negativity (0%), human epidermal growth factor receptor 2 (HER2) negativity (0% by immunohistochemistry), a high proliferative index (Ki-67 >70%), and a programmed death-ligand 1 (PD-L1) combined positive score (CPS) of less than 1. The testing platform is the Department of Pathology at Jilin University Bethune Third Hospital. Results are interpreted according to the grading criteria for Dako 22C3 clone number. Subsequent positron emission tomography-computed tomography (PET-CT) staging revealed stage IV disease (clinical T4N1M1) with lymph node and bone metastases. The patient had no prior medical history or family history of malignancy. Treatment was initiated according to the Chinese Society of Clinical Oncology (CSCO) breast cancer guidelines and consisted of six cycles of capecitabine and paclitaxel liposomes. Serial magnetic resonance imaging (MRI) assessments indicated progressive disease (PD) as the treatment response (**Figure 1**).

Consideration will also be given to changes in the patient’s clinical condition. The patient subsequently underwent six cycles of cadonilimab (10 mg/kg) in combination with gemcitabine(1.4g d1,d8 1/21d) and carboplatin(600mg 1/21d), which were administered every three weeks. During treatment, she experienced grade 3 neutropenia, grade 2 thrombocytopenia, grade 2 nausea, and grade 2 immune-related adverse events, including pruritus and hyperthyroidism.

**Figure 2:**
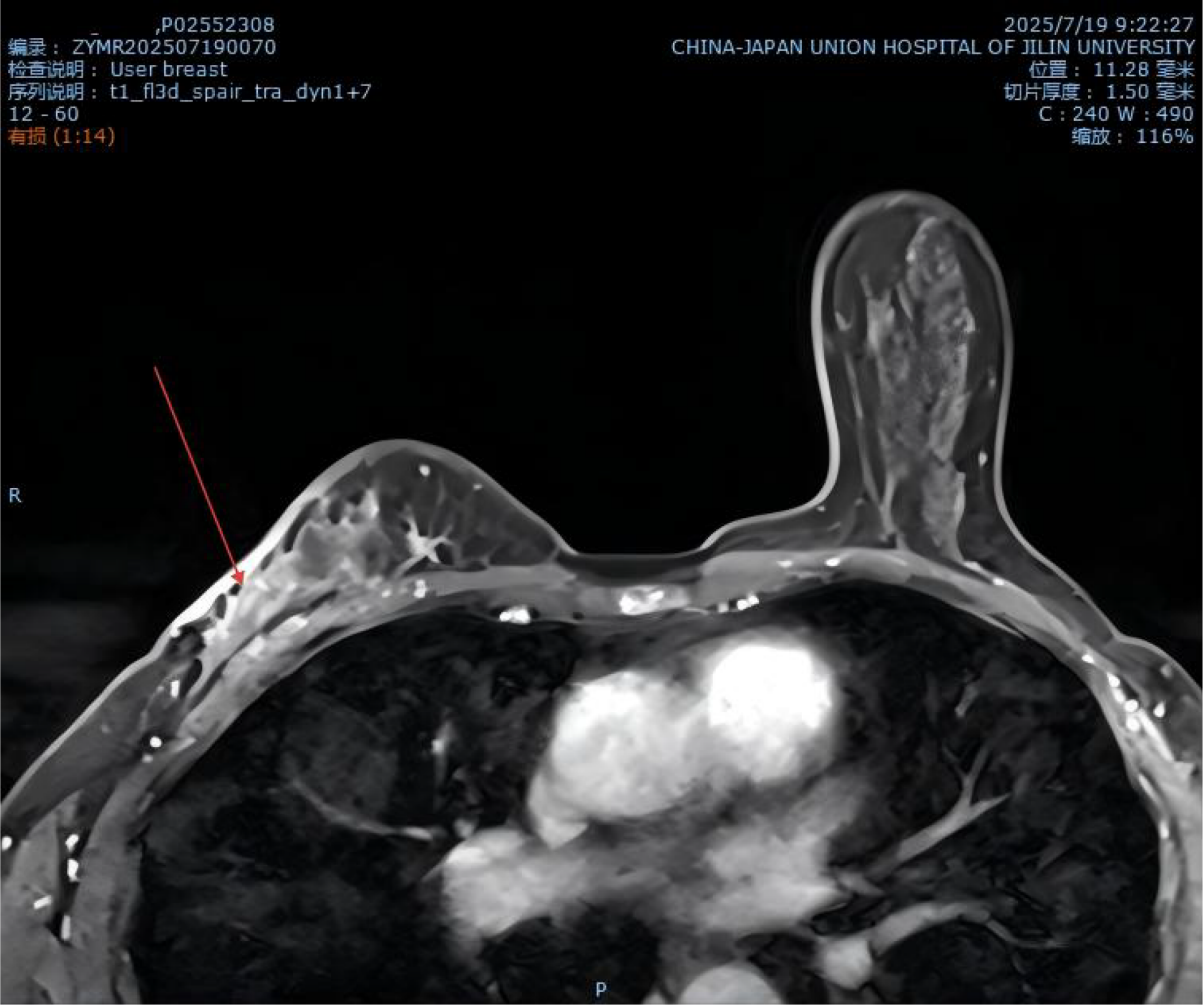
MR image from July 2025.

**Figure 3:**
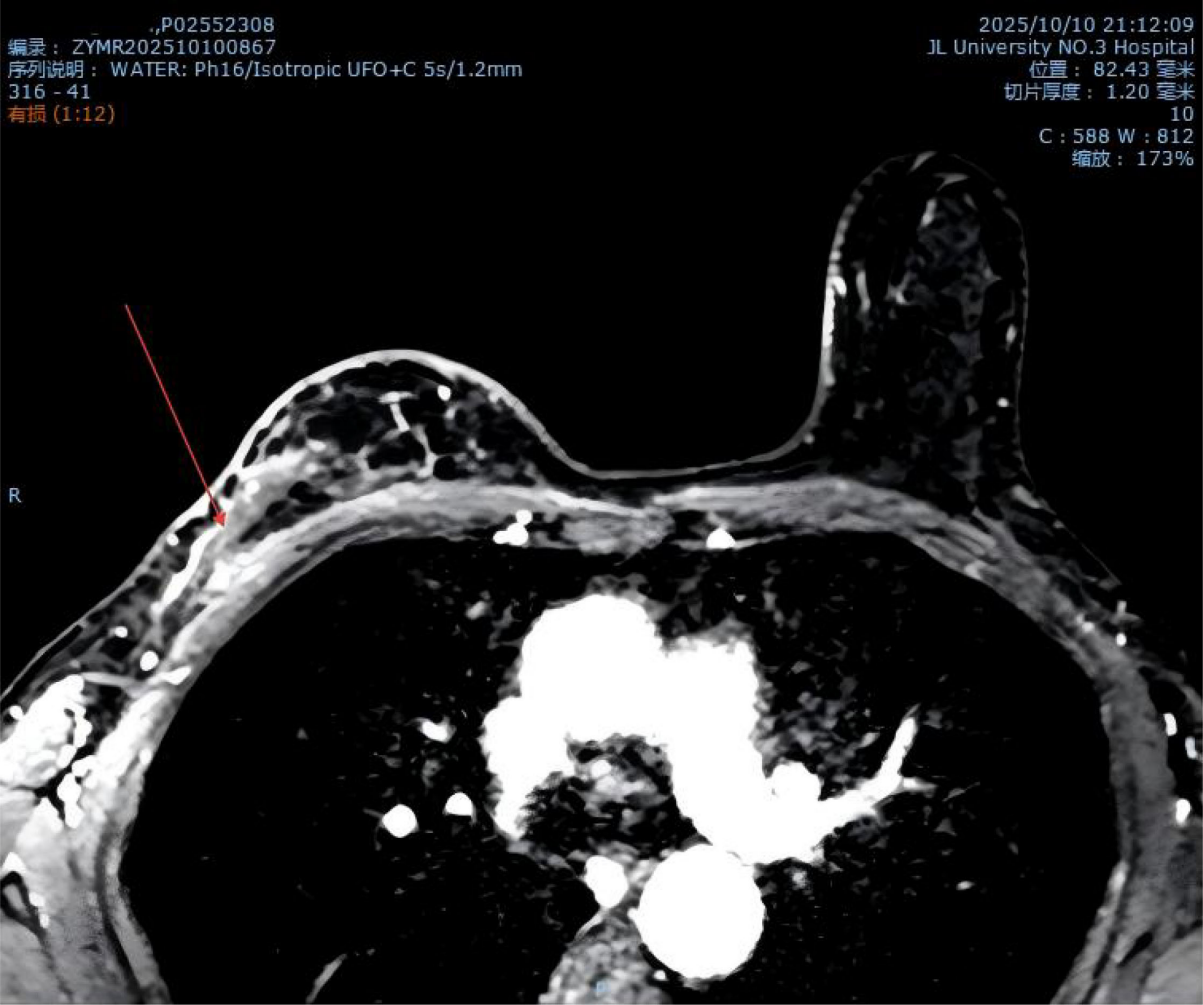
MR image from October 2025.

**Figure 4:**
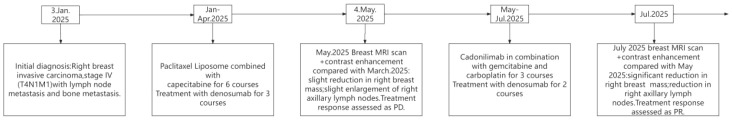
Overall treatment diagram.

In July 2025, the patient underwent both plain and contrast-enhanced breast MRI, which demonstrated a significant PR, as the tumor volume was reduced by more than 75% (**Figure 2**). Based on RECIST criteria, the October 2025 examination results for the target lesion in breast cancer ruled out the possibility of lesion fluctuation (**Figure 3**). The patient’s management trajectory is illustrated in **Figure 4**, outlining key diagnostic milestones and therapeutic interventions (**Figure 4**).

## Discussion

This case highlights that dual-targeted immunotherapy may provide a promising therapeutic option for patients with PD-L1-negative TNBC. In addition, we will examine contemporary therapeutic strategies for the management of TNBC. Although both PD-1 and PD-L1 inhibitors confer survival benefits to TNBC patients, those with PD-L1-positive tumors have the greatest advantage (11). The KEYNOTE-355 trial indicated that the combination of pembrolizumab and chemotherapy yields a significant clinical benefit in terms of PFS among patients with a CPS ≥ 10, whereas no OS benefit was observed in the CPS ≥ 1 subgroup (5). Toripalimab plus nab-paclitaxel tended to improve OS in both the PD-L1 1≤CPS<10 (HR = 0.67, 95% CI: 0.40–1.13) and the CPS ≥ 10 (HR = 0.55, 95% CI: 0.30–0.98) subgroups (6). However, the efficacy of PD-1 or PD-L1 inhibitors is limited in patients who are PD-L1-negative. Key molecular targets for immune checkpoint inhibitors include PD-1, PD-L1, and CTLA-4. In these patients, CTLA-4 inhibitors are increasingly recognized as a crucial component of combination therapy (12).

Compared with ICI monotherapy, combination therapy is superior in terms of OS, the objective response rate (ORR), and PFS (13). In the NCT02834013 study, researchers combined a CTLA-4 inhibitor (ipilimumab) and a PD-1 inhibitor (nivolumab) to treat unresectable or metastatic metaplastic breast cancer. The ORR was 18%; three patients achieved an objective response (OR)[1 complete response (CR) and 2 partial responses (PR)], with a median PFS and OS of 2 months and 12 months, respectively, demonstrating good treatment efficacy. However, 11 patients (65%) experienced adverse events (AEs), including one case of a grade 5 AE. Eight patients (47%) experienced irAEs, with 3 experiencing adrenocortical insufficiency (14). This finding underscores the therapeutic advantages of combination therapy and supports its comparatively reliable safety profile.

Given the advantages of dual targeting and the side effects associated with dual drug administration, cadonilimab—a tetravalent PD-1/CTLA-4 bispecific antibody—has emerged as a preferable option. Approved in 2022 for use in treating various solid tumors, cadonilimab exhibits biological activity comparable to that of combinations of CTLA-4 and PD-1 antibodies (15). Notably, it demonstrates higher binding affinity in environments with a higher PD-1 and CTLA-4 density than in those with a lower PD-1 density, a property not observed with monospecific anti-PD-1 antibodies. Furthermore, cadonilimab does not bind to Fc receptors, resulting in minimal antibody-dependent cytotoxicity, antibody-dependent cellular phagocytosis, and reduced interleukin-6 (IL-6) and IL-8 release. These characteristics likely contribute to the reduced toxicity observed clinically (16). Cadonilimab has been applied to treat various solid tumors, with favorable outcomes (17–20). For instance, in the NCT03872791 trial, the anti-PD-L1/CTLA-4 bispecific antibody KN046 combined with nab-paclitaxel demonstrated promising efficacy as a first-line treatment for metastatic triple-negative breast cancer (TNBC). Among the 25 evaluable patients, the ORR was 44.0% (95% CI: 24.4%–65.1%), the median PFS was 7.33 months (95% CI: 3.68–11.07), and the median OS was 30.92 months (95% CI: 14.75–not estimable) (21). In addition to serving as a cornerstone of conventional chemotherapy-based treatment, immunotherapy is increasingly being integrated with a variety of other therapeutic modalities. Immunotherapy is also combined with other treatment modalities, including radiotherapy and targeted therapies. For example, targeted therapy combined with immunotherapy is clinically employed for TNBC patients harboring BRCA mutations (22). Radiotherapy remains an important component of TNBC treatment (23). A preliminary clinical trial demonstrated that ICIs combined with neoadjuvant stereotactic body radiation therapy (SBRT) can yield favorable outcomes; among 13 enrolled patients, 10 received SBRT, with 90% (9/10) achieving a pathological complete response (pCR), including 3 cases of CR (24). Other immunotherapeutic approaches include personalized peptide vaccines (PPVs), cancer-testis antigens (CTAs), neoantigen vaccines, and RNA vaccines. A PPV-related clinical trial involving 79 patients with metastatic recurrent breast cancer who had failed standard chemotherapy and/or hormone therapy reported a median PFS of 7.5 months and median OS of 11.1 months in patients with metastatic recurrent TNBC (25). Despite these advances, studies specifically investigating cadonilimab in TNBC remain limited.

As research advances, the therapeutic potential of immune checkpoint inhibitors (ICIs) for treating triple-negative breast cancer (TNBC) is becoming increasingly evident. Consequently, identifying which patients are most likely to benefit from such treatments is essential. Predictive biomarkers have garnered considerable attention as tools for screening populations that may respond favorably to ICIs. A study showed that CTLA-4 expression levels emerge as a significant independent factor of good prognosis in BC patients (26).Moreover, cancer-associated fibroblasts (CAFs), tumor-infiltrating lymphocytes (TILs), and α-synuclein (SNCA) have been proposed as immune-related prognostic markers (27). Notably, HIC1 expression is negatively correlated with the tumor mutational burden (TMB) in breast cancer and is elevated in patients who are resistant to pancancer immunotherapy (28).The strong association between CTLA-4 expression and prognosis may be attributable to the favorable therapeutic outcomes observed in patients exhibiting this biomarker profile.

In this case, although the patient’s estrogen receptor (ER), progesterone receptor (PR), and human epidermal growth factor receptor 2 (HER-2) statuses were negative and the programmed death-ligand 1 (PD-L1) combined positive score (CPS) was less than 1, the therapeutic response to cadonilimab was excellent. Recent case reports have demonstrated the efficacy of cadonilimab in treating ovarian cancer, cervical cancer, and lung adenocarcinoma; however, no such reports have been published regarding its use in treating breast cancer (29–31).Previous studies have reported the clinical benefits of cadonilimab in PD-L1-negative ovarian cancer patients (18, 19). Additionally, one study revealed that 4.3% of HER-2-negative patients achieved complete remission (CR) (18).

The therapeutic efficacy is primarily attributed to the combined advantages of CTLA-4 and PD-1/PD-L1 signaling pathway inhibition, with chemotherapy also contributing to the overall effect. The mechanism underlying this combination therapy, which involves two antibodies, includes activation of the immune system to enhance immune responses and inhibit tumor growth. Preclinical studies have demonstrated the synergistic effect of nivolumab and ipilimumab in mouse models (32). Anti-CTLA-4 antibodies function by inhibiting CTLA-4 on the surface of T cells, thereby preventing CTLA-4 from binding to CD80/CD86. This inhibition facilitates the binding of CD28 to CD80/CD86, ultimately leading to T-cell activation. The interaction between PD-L1 and PD-1 promotes immune evasion by inducing T-cell exhaustion and suppressing T-cell function (33). Consequently, PD-1 inhibitors disrupt the PD-1/PD-L1 interaction, thereby enhancing the efficacy of T cells in targeting tumor cells. The combined inhibition of CTLA-4 and PD-1/PD-L1 pathways exerts a synergistic therapeutic effect (34–36). Furthermore, cadonilimab exhibits higher affinity for tumor-infiltrating lymphocytes (TILs) within the tumor microenvironment (TME) than surrounding tissues (37). Combination chemotherapy not only impairs the activity of immunosuppressive cells, such as myeloid-derived suppressor cells (MDSCs) and regulatory T cells (Tregs) but also promotes immune responses by inducing tumor cell apoptosis, enhancing tumor antigen cross-presentation, increasing CD8+ T-cell infiltration, and promoting dendritic cell (DC) maturation (12). In this context, carboplatin was selected for its capacity to directly reduce the tumor burden. Chemotherapy has also been shown to stimulate the release and presentation of tumor antigens, thereby increasing the activation of tumor-infiltrating lymphocytes and contributing to therapeutic efficacy (38–40). In preclinical models, the median survival time with CTLA-4 blockade alone was 25 days, whereas the combination therapy group did not reach this endpoint (P < 0.05). A phase II clinical trial revealed that 16 patients (62%) achieved complete remission, and 6 patients (23%) achieved partial remission. At one year, 58% of patients remained progression-free, with a median follow-up time of 36 months among survivors (41). Therefore, this may help elucidate why dual-targeted immunotherapy combination regimens exhibit enhanced therapeutic efficacy.

In terms of adverse events (AEs), the patient experienced grade 2 immune-related adverse events, specifically pruritus and hyperthyroidism. Elevated serum IL-6 levels have been correlated with increased severity of immune-related cutaneous toxicities, such as maculopapular rash, in patients undergoing immune checkpoint inhibitor therapy (42). Cadonilimab employs an Fc-null design to eliminate Fc-mediated effector functions, thereby preventing the production of proinflammatory cytokines, primarily interleukin-6 (IL-6) and interleukin-8 (IL-8) (3, 43). IL-6 can upregulate the expression of the immune checkpoint molecule PD-L1, which may impact therapeutic efficacy (44, 45). Furthermore, elevated IL-8 expression interferes with PD-L1 blockade and diminishes clinical benefits (46, 47). Therefore, the Fc-null design not only reduces immune-related adverse events but also decreases the production of IL-6 and IL-8, thereby enhancing the efficacy of immune checkpoint inhibitors (ICIs).

It is important to acknowledge several limitations in this case. First, the imaging examination cycle was relatively prolonged, and more comprehensive genetic testing was not conducted. Second, the follow-up period was relatively brief, with insufficient subsequent monitoring to assess the potential for serious adverse reactions. Additionally, there was a lack of further fundamental research to elucidate the underlying reasons for the observed excellent partial response (PR).

In summary, this case illustrates that although the patient was diagnosed with triple-negative breast cancer (TNBC) and had a PD-L1 combined positive score (CPS) of less than 1, highly favorable outcomes were achieved following treatment with cadonilimab, with adverse effects remaining within acceptable and manageable limits. Further large-scale prospective studies are required to substantiate its efficacy and safety, while the potential therapeutic value of multi-targeted immune checkpoint inhibitors in TNBC also merits continued investigation.

## Data Availability

The original contributions presented in the study are included in the article/supplementary material. Further inquiries can be directed to the corresponding authors.
